# Antioxidant and Antiaging Activity of Fermented Coix Seed Polysaccharides on *Caenorhabditis elegans*

**DOI:** 10.3390/nu15112474

**Published:** 2023-05-26

**Authors:** Dan Zhao, Meng Yan, Hualei Xu, Haiyan Liang, Jiachan Zhang, Meng Li, Changtao Wang

**Affiliations:** College of Chemistry and Materials Engineering, Beijing Technology and Business University, Beijing 100048, China; zhao_dan@btbu.edu.cn (D.Z.); yanmeng2019@outlook.com (M.Y.); 2030402111@st.btbu.edu.cn (H.X.); lianghy@th.btbu.edu.cn (H.L.); zhangjiachan@th.btbu.edu.cn (J.Z.); limeng@btbu.edu.cn (M.L.)

**Keywords:** aging, antioxidant, coix seed, fermentation, *Caenorhabditis elegans*, lifespan, IIS pathway

## Abstract

Aging is closely related to many diseases and is a long-term challenge that humans face. The oxidative damage caused by the imbalance of free radicals is an important factor in aging. In this study, we investigate the antioxidant and antiaging activities of fermented coix seed polysaccharides (FCSPs) via in vitro and in vivo experiments. The FCSPs were extracted by fermenting coix seed with *Saccharomyces cerevisiae* for 48 h and utilizing water-extracted coix seed polysaccharides (WCSPs) as a control. Their antiaging activity and mechanism were evaluated based on the antiaging model organism *Caenorhabditis elegans* (*C. elegans*). The results showed that the molecular weight of the FCSPs extracted by fermentation was smaller than that of the WCSPs, making them more easily absorbed and utilized. At a concentration of 5 g/L, the FCSPs’ capacity to scavenge the DPPH·, ABTS^+^·, OH·, and O_2_^−^· radicals was greater than the WCSPs’ capacity by 10.09%, 14.40%, 49.93%, and 12.86%, respectively. Moreover, *C. elegans* treated with FCSPs exhibited higher antioxidant enzyme activities and a lower accumulation of malonaldehyde. By inhibiting the expression of the pro-aging genes daf-2 and age-1, and upregulating the expression of the antiaging genes daf-16, sod-3, skn-1, and gcs-1 in the insulin/insulin-like growth factor-1 (IIS) signaling pathway, the FCSPs could effectively enhance stress tolerance and delay *C. elegans* aging. The lifespan of *C. elegans* in the FCSPs group was 5.91% higher than that of the WCSPs group. In conclusion, FCSPs exert better antioxidant and antiaging effects than WCSPs, which can act as a potential functional ingredient or supplement in food.

## 1. Introduction

The aging of the global population has had a profound impact on the development of society and the economy. The occurrence and development of numerous chronic diseases, such as cardiovascular disease, hypertension, and diabetes, are closely related to aging [1]. The theory of free radical aging notes that aging may result from the accumulation of various active oxygen free radicals in the body [2]. The production and elimination of free radicals in cells are in a dynamic balance. Increasing age and external stimuli can destroy the balance gradually because of an overproduction of free radicals and an impaired ability to neutralize them. Excess free radicals have a strong oxidation ability and can damage the biofilm system and accelerate aging [3]. Supplementing antioxidants can effectively resist the damage of free radicals to the body to delay aging [4]. Natural plant extracts have attracted attention and are widely used in the field of antiaging because of their high safety, significant effect, and low toxic side effects [5].

Coix seed is a homologous drug food in China that has anticancer and antioxidant functions, reduces blood lipids and blood sugar, and supports immunological modulation [6,7,8]. Coix seed extract’s physiological actions are ascribed to several phytochemical components, such as amino acids, vitamins, phenolics, and polysaccharides [9]. Polysaccharides are structural substances and energy sources of plant cells. Plant polysaccharides possess significant bioactivities and physiological functions for humans and are attracting an increasing research interest [10].

Coix seed polysaccharides (CSPs) have many physiological activities, including antioxidant, anti-inflammatory, gut microbiota modification, and immunomodulatory effects [11,12]. The current extraction methods for plant polysaccharides are mainly water extraction [13], ultrasonic extractions [14], and enzymatic extraction [15]. Among these methods, water extraction has the advantages of simplicity and low energy consumption. Still, the excessively high temperature and length extraction procedure can lead to the destruction of the polysaccharide structure, which significantly limits the application of this method [16]. With the extraction of herbal plant components via fermentation technology, the secondary metabolites produced by the microbial metabolism can work together with herbal plant components to achieve a synergistic effect. At the same time, the decomposition and modification of microbial extracellular enzymes promote the release of active ingredients [17,18]. Due to the absence of organic reagents and mild reaction conditions during the extraction process, fermentation is an environmentally friendly method to extract plant polysaccharides and has a strong potential in extracting ingredients [19].

*C. elegans* is a classic model for investigating aging [20]. It has the advantages of easy cultivation, a short growth cycle, highly conservative evolution, and high homology with human genes [21]. Researchers often use *C. elegans* to screen for antiaging natural products and to study aging mechanisms and stress resistance [22,23]. Many signaling pathways are involved in the aging process and conserved between mammals and *C. elegans* [24]. Among these pathways, the IIS signaling pathway is the most important and researched pathway [25]. The IIS pathway can affect the aging process and lifespan of *C. elegans* by regulating multiple genes. These genes include cellular stress and metabolic genes, and specific lifespan-shortening genes [26]. Daf-16 is a crucial regulator of *C. elegans* aging and aging-related biological processes in the IIS pathway [27]. Apart from daf-16, skn-1 plays a significant role in extending the lifespan of *C. elegans* as another prominent direct target of IIS [28,29].

**Figure 1:**
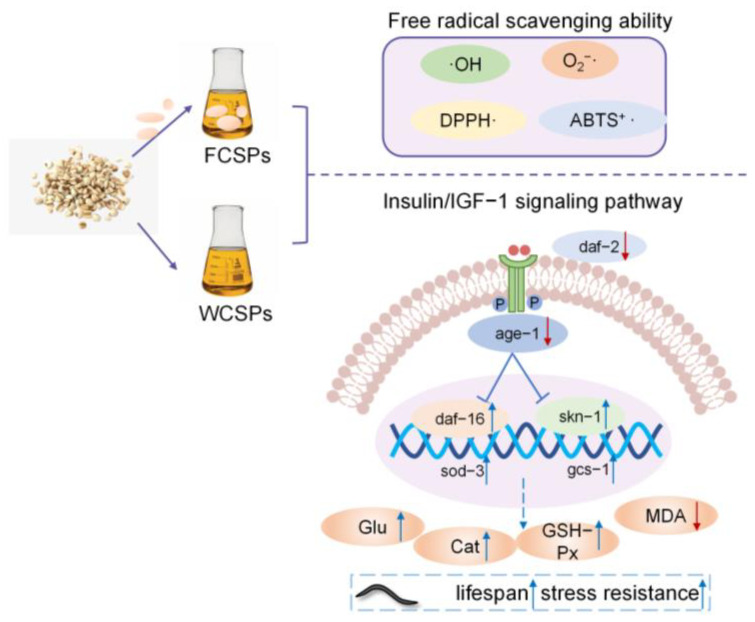
Schematic diagram showing the antiaging mechanism of WCSPs and FCSPs at the in vitro and in vivo levels.

Previous studies have shown that the lipophilic antioxidant compounds are increased after the fermentation of coix seeds by *Monascus purpureus* [30]. It was also reported that the fermentation of coix seeds with *Lactobacillus plantarum* could improve their nutritional and stability properties [31]. After fermentation with *Sanghuangporus sanghuang*, the antioxidant capacity of coix seeds was enhanced [32]. Although the benefits of fermented coix seed were reported, few studies have investigated the antioxidant and antiaging effects of FCSPs. The purpose of this article is to evaluate the antiaging activity and mechanisms of FCSPs using *C. elegans* as an aging model organism. As shown in Figure 1, we first fermented coix seeds with *Saccharomyces cerevisiae* (*S. cerevisiae*) to obtain FCSPs. Simultaneously, WCSPs were prepared as the control. Then, we explored the free radical scavenging ability and antiaging effects of FCSPs and WCSPs from in vitro and in vivo perspectives. Moreover, the antiaging mechanism of FCSPs was investigated by analyzing aging-related genes in the IIS signaling pathway in *C. elegans*.

## 2. Materials and Methods

### 2.1. Materials

*S. cerevisiae* was purchased from the China General Microbiological Culture Collection Center, and coix seed was produced in Guizhou, China. *E. coli* OP50 and *C. elegans* were preserved in the laboratory. The catalase (CAT) assay kit, total superoxide dismutase (SOD) assay kit with the NBT method, glutathione peroxidase (GSH-Px) assay kit, and lipid peroxidation malondialdehyde (MDA) assay kits were obtained from Beyotime, China. cDNA Synthesis SuperMix and TransStart^®^ Top Green qPCR SuperMix were purchased from TransGen Biotech, Beijing, China. All the other chemical reagents were analytically pure.

### 2.2. Sample Preparation

Coix seeds were pulverized with a high-speed multifunctional pulverizer and passed through a 50-mesh sieve to obtain coix seed powder. For the preparation of WCSPs, 8 g of coix seed powder was added to 200 mL of deionized water and autoclaved at 121 °C for 30 min and cultured in a 37 °C incubator with shaking for 48 h [33]. For the preparation of FCSPs, 8 g of coix seed powder was added to 200 mL of deionized water and autoclaved at 121 °C for 30 min. *S. cerevisiae* was inoculated into the solution and cultured in a 37 °C incubator with shaking for 48 h. 

Then, the water extract and fermentation broth were centrifuged at 5000 rpm for 10 min, and the supernatant was enzymatically hydrolyzed with α-amylase and papain for 3 h. The solution was then fully mixed with 3 times the volume of anhydrous ethanol and placed overnight at 4 °C. The supernatant was removed, and the residue was redissolved in distilled water and precipitated with anhydrous ethanol. The final sediment was freeze-dried to obtain WCSPs and FCSPs.

### 2.3. Molecular Weight Determination of Polysaccharides

A total of 0.3 g of the sample was added to a 5 mL sample bottle, ultrapure water containing 0.1 mol/L sodium nitrate was added to reach 5 mL, and the sample was ultrasonically dispersed before testing. The mobile phase was ultrapure water containing 0.1 mol/L sodium nitrate and the flow rate was 1 mL/min. The standard substance was narrow-distribution polyethylene glycol with molecular weights 330,000, 176,000, 82,500, 44,000, 25,300, 20,600, 12,600, 7130, 4290, 1400, 633, and 430. The GPC analysis conditions were as follows: Waters 1515 isocratic HPLC pump; ULTRAHYDROGEL 250 PKGD column; Waters 2414 refractive index detector. 

### 2.4. Analysis of Polysaccharide Components

To hydrolyze polysaccharides, 2 mg of purified polysaccharides was added to 5 mL of 2 mol/L trifluoroacetic acid (TFA), purged with nitrogen to remove the air, hydrolyzed at 120 °C for 4 h, and combined with anhydrous methanol. The mixture was subjected to repeated rotary evaporation to remove TFA and finally dissolved in 2 mL of deionized water. A total of 100 µL of this mixture was weighed into a new centrifuge tube, and deuterium-labeled succinic acid (10 µL, 1.5 mg/mL) was added; the mixture was vacuum freeze-dried, combined with 50 L of 20 mg/mL methoxyammonium hydrochloride/pyridine solution reacted in a water bath at 40 °C for 80 min, combined with 80 μL of *N*-methyl-*N*-(trimethylsilyl) trifluoroacetamide, incubated at 40 °C for 80 min, and then centrifuged at 12,000 rpm. The supernatant was filtered with a membrane, placed into a sample bottle, and left to stand at room temperature for 2 h before performing GC-MS analysis.

### 2.5. Antioxidant Ability Detection In Vitro

#### 2.5.1. Detection of the DPPH Radical (DPPH·) Scavenging Rate

WCSPs and FCSPs were diluted with deionized water to different concentrations. Three milliliters of the sample was reacted with DPPH solution of the same volume in the dark for 30 min, and the absorbance was measured at 517 nm as A_1_. The absorbances of deionized water mixed with DPPH solution and deionized water mixed with samples were set as A_2_ and A_3_, respectively. The DPPH· scavenging ability was calculated as follows: DPPH· scavenging ability (%) = (A_2_ + A_3_ − A_1_)/A_2_ × 100% [34].

#### 2.5.2. *Detection of the ABTS Radical (ABTS*^+^*·) Scavenging Rate*

FCSPs and WCSPs were dissolved into different concentrations with deionized water. The ABTS stock solution was prepared by adding 0.89 mL of 140 mmol/L potassium persulfate solution to 50 mL of 7 mmol/L ABTS radical solution and stored at 4 °C overnight. Before use, ABTS stock solution was diluted to an absorbance value of 0. 70 ± 0. 02 at 734 nm to make ABTS working solution. Then, 1.9 mL of ABTS working solution was mixed evenly with 0.1 mL of sample solution and incubated at room temperature for 6 min. The absorbance at 734 nm was determined. A_1_ is the absorbance of ABTS working solution and sample solution; A_2_ is the absorbance of replacing equal volume ABTS working solution with deionized water; and A_0_ is the absorbance of replacing equal volume sample solution with deionized water. ABTS radical scavenging rate (%) = (1 − (A_1_ − A_2_)/A_0_) × 100% [35].

#### 2.5.3. Detection of the Hydroxyl Radical (·OH) Scavenging Rate

First, 0.5 mL of 0.75 mmol/L o-phenanthroline absolute ethanol solution was successively mixed with 1 mL of 0.15 mol/L PBS and 0.5 mL of distilled water. Then, 0.5 mL of 0.75 mmol/L ferrous sulfate solution was added and mixed fully. Finally, 0.5 mL of 0.01% hydrogen peroxide was added and incubated at 37 °C for 1 h. The absorbance was measured at 536 nm, and the obtained value was A_1_. The 0.5 mL of 0.01% hydrogen peroxide in tube A_1_ was replaced with 0.5 mL of distilled water, and the other steps were the same as for A_1_ to obtain the absorbance value A_2_. For the sample tube, 0.5 mL of distilled water in A_1_ was replaced with 0.5 mL of sample, and the other steps were the same as for A_1_; the absorbance value was named A_3_. Hydroxyl radical scavenging rate (%) = (A_3_ − A_1_)/(A_2_ − A_1_) × 100% [36].

#### 2.5.4. Detection of the Superoxide Anion (O^2−^·) Scavenging Rate

First, 2.25 mL of 0.05 mol/L Tris-HCl (pH 8.2) buffer solution was added to each tube and placed in a 25 °C water bath to preheat for 20 min. Then, 0.05 mL of sample solution and 0.2 mL of 25 mmol/L pyrogallol solution were mixed evenly and reacted in a water bath at 25 °C for 5 min. The reaction was terminated by adding 0.5 mL of 8 mol/L hydrochloric acid, and the absorbance at 299 nm was measured as A_1_. In the blank control group, 0.05 mL of sample solvent was used to replace the sample, and the absorbance was A_2_. Superoxide anion scavenging rate (%) = (A_2_ − A_1_)/A_2_ × 100%, where A_1_ is the average absorbance of the blank and A_2_ is the average absorbance of the sample [36].

### 2.6. Antiaging Activity Detection In Vivo

#### 2.6.1. Culture and Synchronization of *C. elegans*

NGM solid medium was prepared in sterile culture dishes. After solidification, 200 μL of *E. coli* OP50 bacteria solution was added to the NGM plate and spread evenly. The plate was incubated at 37 °C overnight. *C. elegans* in the L4 stage were transferred to NGM plates and cultured at 20 °C. To cultivate synchronized *C. elegans*, the nematode in a good growth state was selected and collected into centrifuge tubes with 4.5 mL of sterile water. A total of 2 mL of lysate was added, the mixture was vortexed every 2 min, and the process was repeated 4–6 times until the *C. elegans* were lysed entirely. The lysed mixed solution was transferred to a 1.5 mL centrifuge tube and centrifuged at 3000 rpm for 1 min, and the supernatant was discarded. Then, sterile water was added to wash the *C. elegans*, the supernatant was discarded after centrifugation, and this procedure was repeated 3 times. The eggs were collected from the bottom of the centrifuge tube, dropped onto the sterile area of the NGM plate coated with *E. coli* OP50, and dried. After 48 h of cultivation at 20 °C, the fertilized eggs developed into the L4 stage of *C. elegans* and were used for subsequent experiments.

#### 2.6.2. Effects of WCSPs and FCSPs on the Longevity of *C. elegans*

Plates containing samples with different concentrations were prepared so that the final concentrations of WCSPs and FCSPs in *E. coli* OP50 bacterial liquid were 0.2, 1, and 5 g/L, and the control group was the M9 buffer. The synchronized *C. elegans* were transferred to culture medium containing samples at 20 °C, with 30 *C. elegans* specimens per plate and three parallels for each group. The *C. elegans* were transferred to a new corresponding Petri dish every day, and the number of live and dead *C. elegans* specimens was recorded until all *C. elegans* were dead. If the *C. elegans* specimens did not respond to stimulation twice, they were determined to be dead. The *C. elegans* that escaped, dried out, and had eversion of the reproductive tract was eliminated. The lifespan curve of the *C. elegans* was generated according to the data recorded in the experiment.

#### 2.6.3. Effects of WCSPs and FCSPs on the Oviposition Ability of *C. elegans*

The culture medium was prepared so that the final concentration of WCSPs and FCSPs in the *E. coli* OP50 bacterial solution was 5 g/L (the optimal concentration screened out by the lifespan test), and the control group was the M9 buffer. The synchronized *C. elegans* were placed on a plate, with one *C. elegans* specimen per plate and a total of 10 parallels, and cultured at 20 °C. The *C. elegans* were transferred to a new plate each day until the end of the oviposition period. All eggs were placed in a constant temperature incubator at 20 °C. After 2 days, the number of larvae in each plate was calculated [37].

#### 2.6.4. Effects of WCSPs and FCSPs on the Anti-Heat Stress Ability of *C. elegans*

The culture medium was prepared so that the final concentration of WCSPs and FCSPs in the *E. coli* OP50 bacterial solution was 0.2, 1, and 5 g/L, and the control group was the M9 buffer. Synchronized *C. elegans* were transferred onto plates with 30 *C. elegans* specimens per plate and cultured at 20 °C. Three repetitions were made for each treatment. *C. elegans* were moved to a new Petri dish each day for 5 days. The plates were then transferred to a 35 °C incubator for cultivation, and the start time was 0 h. The number of surviving and dead specimens were recorded every 2 h until all *C. elegans* died. The lifespan curve was plotted according to the survival rate of nematodes at different time periods [27].

#### 2.6.5. Effects of WCSPs and FCSPs on the Antioxidant Stress Ability of *C. elegans*

The culture medium containing WCSPs and FCSPs was prepared so that the final concentration in the *E. coli* OP50 bacterial solution was 0.2, 1, and 5 g/L, and the control group was the M9 buffer. Synchronized *C. elegans* were transferred onto plates with 30 specimens per plate and cultured at 20 °C. Three repetitions were made for each treatment. *C. elegans* were shifted to a new Petri dish every day for five consecutive days. They were then moved to an NGM medium containing 500 μmol/L juglone and incubated at 20 °C. The number of dead and surviving *C. elegans* was observed and recorded every 2 h until all nematodes were dead [22]. The lifespan curve was plotted according to the survival rate of nematodes at different time periods.

#### 2.6.6. Detection of Antioxidant Enzyme Activity and MDA Content

Synchronized L4 stage *C. elegans* were cultured with FCSPs at different concentrations for 5 days, washed with M9 buffer, collected in sterile enzyme-free EP tubes, and suspended in 1 mL of lysis buffer. The suspension was homogenized via ultrasonication in an ice bath, followed by centrifugation at 6000 rpm for 10 min at 4 °C. The MDA content and antioxidant enzyme activity (including SOD, CAT, GSH-Px) were determined according to the kit instructions. The specific steps were the same as those described in the kit, and each experiment was repeated thrice. The absorbance was measured using microplate reader (Thermo Fisher Scientific, Waltham, MA, USA). 

#### 2.6.7. Gene Detection via qRT-PCR

Synchronized L4 stage *C. elegans* were cultured with various concentrations of FCSPs and moved to a new plate every day. After 5 days of culture, they were washed with M9 buffer and collected in sterile enzyme-free EP tubes. The worms were centrifuged at 3500 r/min for 1 min, the supernatant was discarded, and *C. elegans* specimens were re-suspended in M9 buffer. The centrifuge protocol was repeated three times. After quick freezing in liquid nitrogen, *C. elegans* RNA was extracted using the TRIzol method. The cDNA first-strand synthesis kit was used to reverse-transcribe the RNA into cDNA and perform fluorescence quantitative detection (QuantStudio 3, Thermoscientific, Shanghai, China). The amplification conditions were 95 °C for 3 min, then 95°C for 30 s, 62 °C for 40 s, and 72 °C for 50 s, for a total of 40 cycles. The primer sequences are listed in Table 1.

### 2.7. Statistical Analysis

Antioxidant and antiaging experiments were performed three times separately. Oviposition capacity was determined via 10 replicate experiments. Data were expressed as mean ± standard deviation (SD). Statistical analysis was performed using IBM SPSS 22 software. The normality of the data was first determined by ANOVA. For data with normality, differences between the groups were analyzed by ANOVA. Non-parametric analysis via Mann–Whitney U was used to compare data that did not have normality. Data with * *p* < 0.05 and ** *p* < 0.01 were considered statistically significant and extremely significant, respectively.

## 3. Results

### 3.1. Polysaccharide Composition of WCSPs and FCSPs

**Figure 2:**
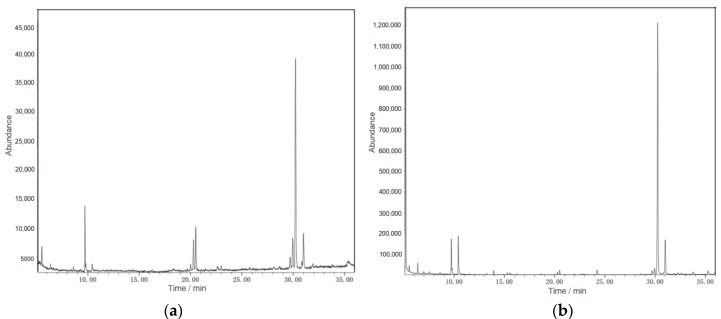
GC-MS spectra of (**a**) WCSPs and (**b**) FCSPs.

In this paper, water extraction and fermentation methods were used to extract the WCSPs and FCSPs, and the polysaccharide components were analyzed via GC-MS (Figure 2). Both the WCSPs and FCSPs were composed of five monosaccharides, as shown in Table 2. The five monosaccharides included xylose, arabinose, mannose, galactose, and glucose. Among them, glucose accounted for the most significant molar percentage, which increased from 68.06% to 93.04% after fermentation. The difference in the monosaccharides’ composition could be brought on by microbial fermentation, which transforms the CSPs into oligo- or monosaccharides [23].

### 3.2. Molecular Weight Detection

**Figure 3:**
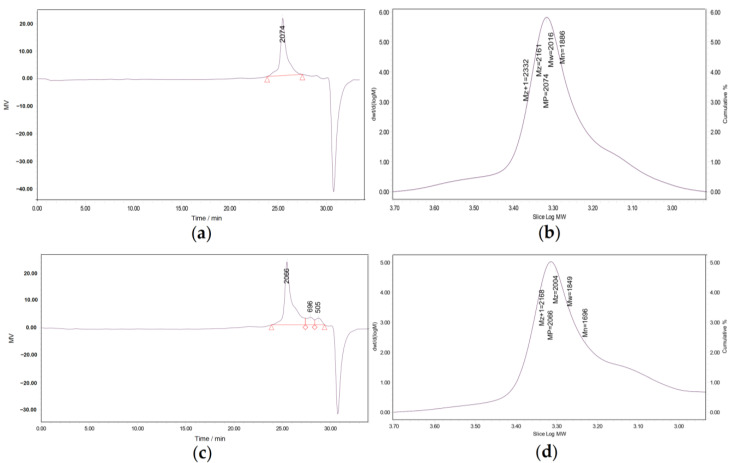
Autoscaled chromatograms and GPC calibration spectra of WCSPs (**a**,**b**) and FCSPs (**c**,**d**).

The molecular weight of polysaccharides and their bioactivity are closely associated. Polysaccharides with large molecular weights and poor water solubility are not conducive to absorption in the body, and their biological activity is reduced, which may limit the application of polysaccharides, while low-molecular-weight polysaccharides less than 10 kDa have a relatively high activity on human health [38]. As shown in Figure 3, GPC was used to characterize the molecular weight of the polysaccharides. The results show that the Mw molecular weight of the WCSPs was 2016 Da, while the Mw of the FCSPs was 1849 Da. After fermentation, the Mw of the CSPs decreased, which was due to the enzyme produced by microorganisms hydrolyzing the polysaccharides into those with lower molecular weights [39]. The Mw/Mn of the WCSPs and FCSPs was 1.06 and 1.09, respectively, indicating the good polydispersity of the two extracts, and the distribution of the molecular chain length was uniform.

### 3.3. Antioxidant Ability Detection In Vitro

**Figure 4:**
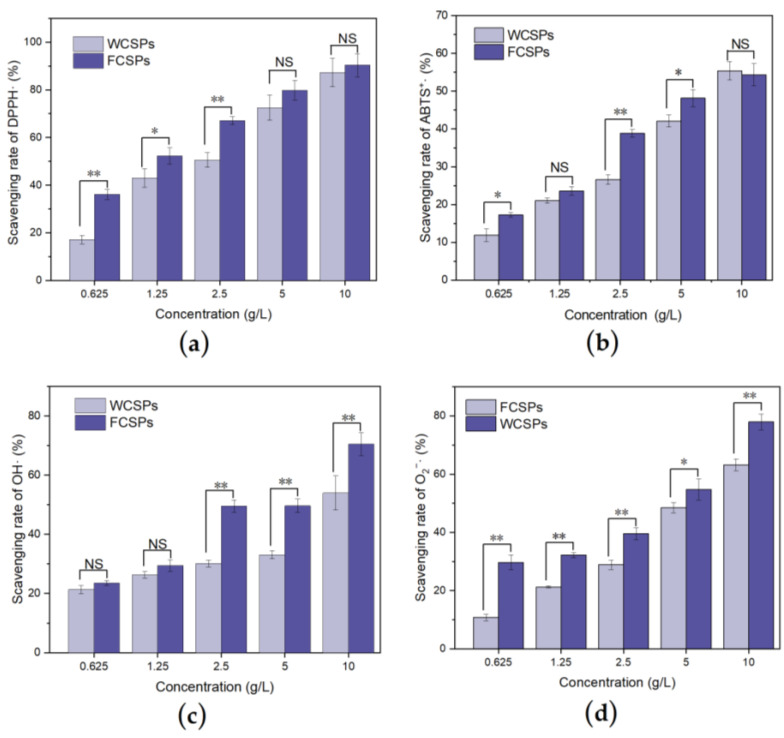
Effect of WCSPs and FCSPs on the free radical scavenging rate. (**a**) DPPH·, (**b**) ABTS^+^·, (**c**) OH·, and (**d**) O_2_^−^. Compared between the two groups, NS indicates no significant difference, * *p* < 0.05; ** *p* < 0.01.

The polysaccharides of many plants, such as oats, wolfberry, and white fungus, have antioxidant properties [40,41,42]. Natural antioxidants can delay the aging caused by oxidative stress damage by inhibiting the production of free radicals or slowing the oxidation reaction. The antioxidant activity of different concentrations of WCSPs and FCSPs was tested in vitro. As shown in Figure 4, both the WCSPs and FCSPs had a certain scavenging effect on the DPPH·, ABTS^+^·, OH·, and O_2_^−^· radicals, and the relationship was dose-dependent. Among them, the IC_50_ values of the WCSPs for scavenging the DPPH·, ABTS^+^·, OH· and O_2_^−^· were 2.83, 27.04, 11.64, and 5.63 g/L, respectively, and the IC_50_ values of the FCSPs for scavenging the above four free radicals were 1.41, 6.39, 3.53, and 3.03 g/L, respectively, which was nearly half the dose value of the WCSPs, so the antioxidant activity of the FCSPs was stronger. At a concentration of 5 g/L, the scavenging capacities of the FCSPs for the DPPH·, ABTS^+^·, OH·, and O_2_^−^· radicals were 10.09%, 14.40%, 49.93%, and 12.86% higher than those of the WCSPs, respectively.

### 3.4. Antiaging Activity on C. elegans

#### 3.4.1. Lifespan and Oviposition Ability of *C. elegans*

**Figure 5:**
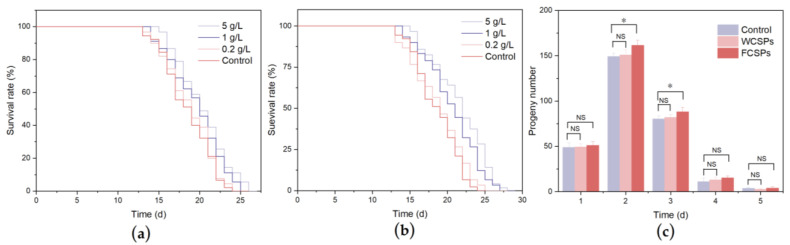
Effect of WCSPs (**a**) and FCSPs (**b**) on the lifespan of *C. elegans*. (**c**) Effect of WCSPs and FCSPs on the oviposition ability of *C. elegans*. Compared with the control group, NS indicates no significant difference, * *p* < 0.05.

The effect of CSPs on the lifespan of *C. elegans* was investigated via treatment with different concentrations of WCSPs and FCSPs. As shown in Table 3, the average lifespan of the control group was 18.02 ± 1.71 days. The survival curve of *C. elegans* shifted to the right as the concentrations of the WCSPs and FCSPs increased (Figure 5a,b). After intervention with 5 g/L of WCSPs, the average lifespan of *C. elegans* was 21.32 ± 1.08 days, which was 18.31% higher than that of the control group. For the FCSPs treatment group, the lifespan extension effect was more significant, as the average lifespan was 22.58 ± 1.21 days, and the corresponding increase was 25.30%. The lifespan of *C. elegans* in the FCSPs group was 5.91% higher than that of the WCSPs group. As a model organism of aging, the length of the *C. elegans* lifespan can be an important indicator of aging. The results showed that both the FCSPs and WCSPs could prolong the lifespan of *C. elegans* in a dose-dependent manner. Compared with the WCSPs, the FCSPs had a better effect on extending the lifespan and delaying the aging of *C. elegans*.

The oviposition ability of *C. elegans* is related to its growth and development status. As high doses of WCSPs and FCSPs could significantly delay the aging of *C. elegans*, 5 g/L was selected as the treatment concentration to investigate whether it negatively impacted *C. elegans* reproduction. As shown in Figure 5c, the total number of eggs produced by the control group was 293.02 ± 13.34, and the accumulated number of eggs laid by the WCSPs group was 297.80 ± 10.62. The number of eggs was increased by 1.63% compared to the control group, but the change was not significant. After FCSPs treatment, the total egg number was 320.2 ± 9.19, which was significantly increased in comparison with the control group, and the increase rate was 9.28% (*p* < 0.05). Previous studies show that lifespan is positively related to reduced reproduction [43], but the WCSPs and FCSPs could extend the lifespan of *C. elegans* without damaging their reproductive ability, indicating that WCSPs and FCSPs at 5 g/L can exert their antiaging effect while being safe.

#### 3.4.2. Stress Resistance of *C. elegans*

**Figure 6:**
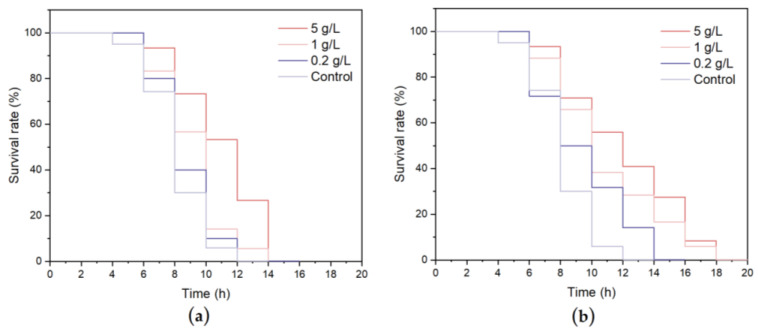
Effects of WCSPs (**a**) and FCSPs (**b**) on the survival rate of *C. elegans* under 35 °C heat stress.

One of the mechanisms for extending lifespan is the improvement of the resistance of cells to stress conditions, so the extension of lifespan is highly correlated with the survival rate under stress conditions [44]. As shown in Figure 6 and Table 4, under the heat stress condition of 35 °C, the average lifespan was 8.10 ± 0.37 days in the control group, and the lifespan curves of the *C. elegans* treated with WCSPs and FCSPs shifted significantly to the right. The 5 g/L WCSPs and FCSPs treatments had the best protection against heat stress, with lifespans of 10.93 ± 0.69 days and 11.21 ± 0.71 days, respectively, which were 34.93% and 38.93% higher than the control group (*p* < 0.01). Therefore, WCSPs and FCSPs can improve the heat tolerance of *C. elegans* and delay aging. 

Improving the ability to resist oxidative stress is the primary indicator of biological resistance to aging. To explore the effects of WCSPs and FCSPs on *C. elegans* oxidative stress damage, the *C. elegans* treated with samples for five days were transferred to NGM plates with 500 μmol/L juglone for culture, and the survival rate of the *C. elegans* was observed and calculated.

**Figure 7:**
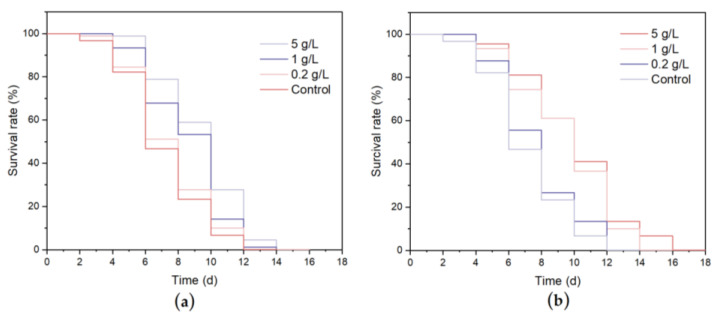
Effects of WCSPs (**a**) and FCSPs (**b**) on the survival rate of *C. elegans* under oxidant stress.

As shown in Figure 7 and Table 5, the average lifespan of the control group was 7.04 ± 0.17 h under oxidative stress. When treated with 0.2, 1, and 5 g/L WCSPs, the average lifespan increased in a dose-dependent manner to 7.42 ± 0.41 h, 8.59 ± 0.22 h, and 8.59 ± 0.22 h, corresponding to increases of 5.39%, 22.01%, and 33.23%, respectively, compared to the control group. The average lifespan of the *C. elegans* treated with 0.2, 1, and 5 g/L FCSPs increased by 8.94%, 35.08% and 39.77%, respectively. The FCSPs treatment group had more robust resistance to oxidative damage than the WCSPs treatment group. Therefore, WCSPs and FCSPs can increase the survival time of *C. elegans* under standard or stress conditions, which may be due to the antioxidant activity of CSPs. To further explore the mechanism of the antioxidant effect of WCSPs and FCSPs, the actions of related antioxidant enzymes in *C. elegans* were analyzed.

#### 3.4.3. Antioxidant Enzyme Activities and MDA Content Detection in *C. elegans*

**Figure 8:**
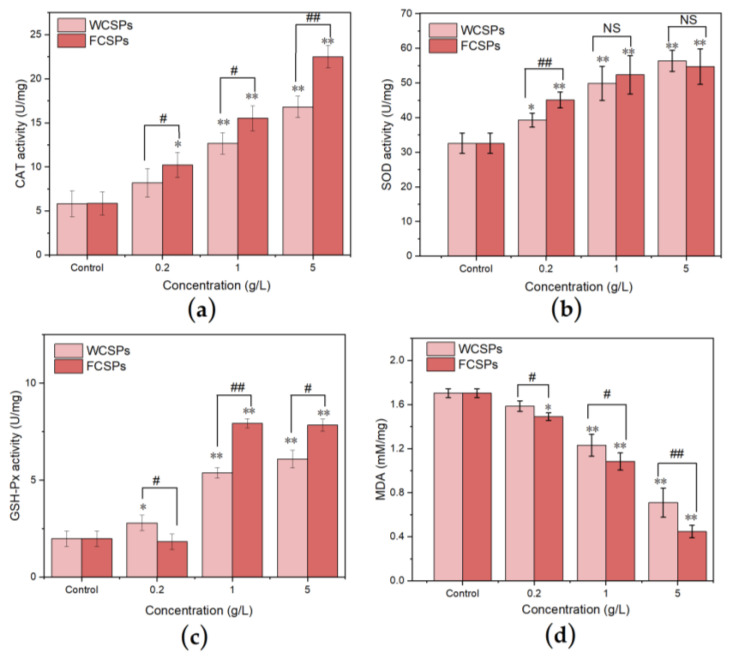
Effects of WCSPs and FCSPs on the CAT activity (**a**), SOD activity (**b**), GSH-Px activity (**c**), and MDA content (**d**) of *C. elegans*. Compared with the control group, * *p* < 0.05; ** *p* < 0.01. Compare between the two groups, NS indicates no significant difference, ^#^ *p* < 0.05; ^##^ *p* < 0.01.

Under normal conditions, free radicals in the body are in a dynamic balance, and oxidative stress and antioxidant defense are also in a dynamic balance. The body’s production of antioxidant enzymes decreases as it ages, which disrupts the dynamic balance and accelerates aging [4]. SOD is a superoxide radical scavenging factor naturally present in the body that can convert harmful superoxide radicals into hydrogen peroxide through a disproportionation reaction. CAT and GSH-Px in the body immediately decompose radicals into water and oxygen, and the three enzymes form a complete antioxidation chain [45]. The activities of the three antioxidant enzymes in the *C. elegans* treated with WCSPs and FCSPs were positively correlated with an increase in the treatment concentration. At concentrations of 0.2 g/L, 1 g/L, and 5 g/L, the CAT and SOD enzyme activities of the FCSP treatment group were significantly higher than those of the WCSPs group. The FCSPs significantly increased the activity of the GSH-Px enzyme at moderate and high doses of 1 g/L and 5 g/L. MDA is a lipid peroxidation product formed by intracellular ROS attacking polyunsaturated fatty acids in biomembranes. The amount of MDA can usually reflect the degree of lipid peroxidation in the body and indirectly reflect the degree of cell damage [46]. Figure 8 shows that the MDA content in the *C. elegans* that were fed FCSPs and WCSPs significantly decreased, indicating that both kinds of CSPs could reduce free radical attack in *C. elegans*.

#### 3.4.4. Effect of FCSPs on the Gene Expression of *C. elegans*

**Figure 9:**
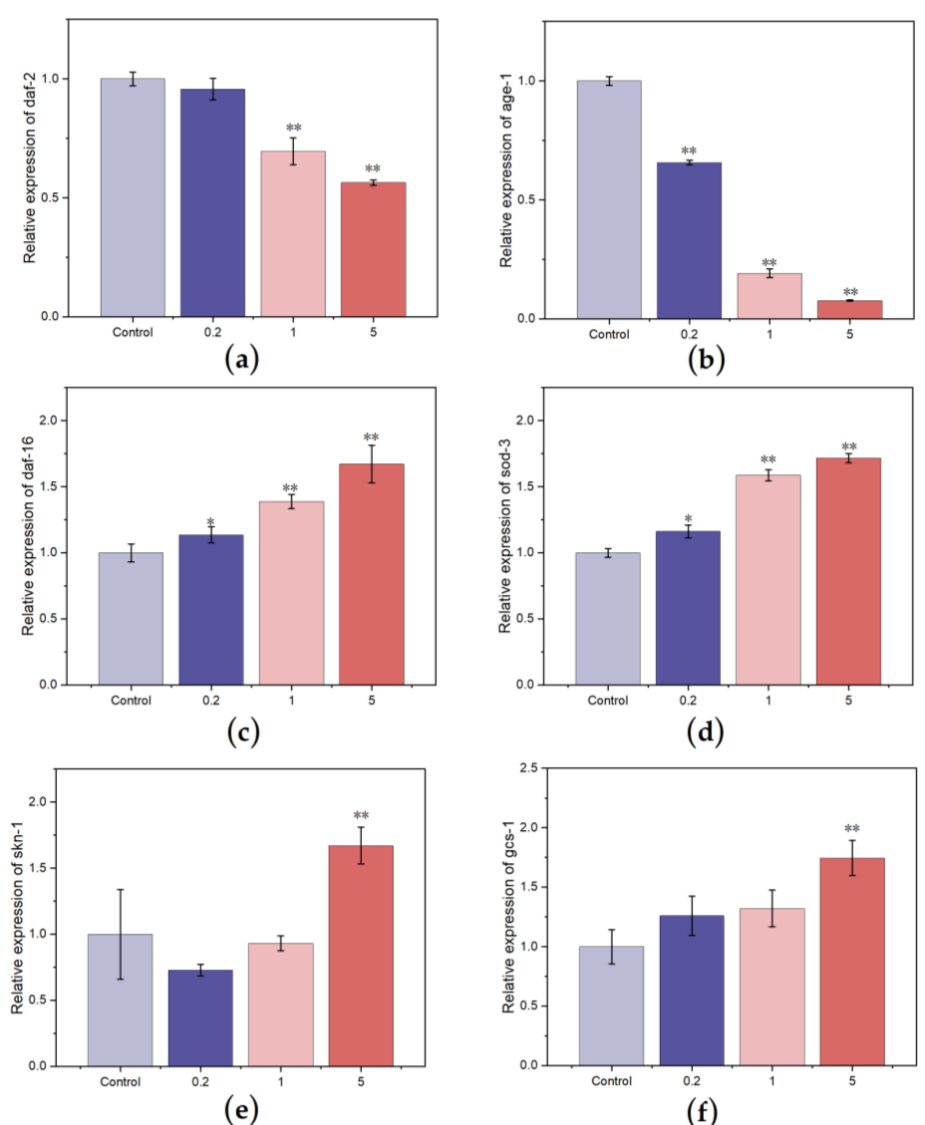
Effect of FCSPs on the gene expression of *C. elegans*. (**a**) Daf-2, (**b**) age-1, (**c**) daf-16, (**d**) sod-3, (**e**) skn-1, and (**f**) gcs-1. Compared with the control group, * *p* < 0.05; ** *p* < 0.01.

The IIS pathway plays an important role in regulating *C. elegans* aging. The daf-2 gene in this pathway is a crucial gene of the *C. elegans* senescence. Daf-2 affects lifespan by downregulating the expression of daf-16 during *C. elegans* development [47,48]. Sod-3 is a downstream gene of daf-16. When *C. elegan*s is stimulated by external stresses, such as heat and oxidative environments, DAF-16 translocates to the nucleus and thereby upregulates its downstream effector elements to improve stress resistance [49]. The IIS pathway contains many components that regulate aging, and the age-1 gene is the main upstream component that regulates various physiological factors, including aging and lifespan. The inhibition of the age-1 gene can significantly extend the lifespan of *C. elegans* [50]. Figure 9 shows that the *C. elegans* that were administered FCSPs can significantly reduce the upstream genes daf-2 and age-1 of IIS, and the expression of daf-16 was upregulated. In addition, the RT-PCR results showed that the 0.2, 1, and 5 g/L FCSPs treatment groups could significantly upregulate the expression of sod-3, which is the target gene of daf-16. SKN-1 and DAF-16 play key roles in maintaining a long lifespan. SKN-1 is a homologous protein of the mammalian Nrf2 protein, which functions as a transcription factor [51]. SKN-1 plays an important role in physiological stress, detoxification, oxidation balance regulation, immune and lipid metabolism regulation, etc. It is an essential regulatory factor required for the life extension of some mutants. Gcs-1 is a downstream gene of skn-1, encodes glutamylcysteine synthetase, and participates in the biosynthesis of glutathione [52]. Real-time PCR results showed that skn-1 and its target gene gcs-1 were increased by 67.1% and 74.6%, respectively, after treating *C. elegans* with FCSPs.

The above results indicate that FCSPs could delay the aging of *C. elegans* by regulating two key routes, daf-16 and skn-1, in the IIS pathway to jointly affect the antioxidant and stress tolerance of *C. elegans*.

## 4. Discussion

Coix seed is a traditional Chinese medicinal and edible plant that is widely used in food and health products. Studies have shown that coix seeds contain a variety of active ingredients and have antioxidant, immunoregulatory, and antiaging effects [53,54]. In this paper, we used the fermentation method to extract CSPs. This method has mild extraction conditions and can reduce the loss of active ingredients caused by high temperatures. Fermentation technology utilizes the enzymes metabolized by microorganisms to decompose substrates while producing other substances [17]. The results showed that the molecular weight of the CSPs decreased, and the polysaccharide composition also changed. The change in the molecular weight was in accordance with the work of Wang et al. [37], who extracted *Lycium barbarum* polysaccharides with lower molecular weights via fermentation. The CSPs were decomposed by *S. cerevisiae* during the fermentation process, producing polysaccharides with reduced molecular weights that facilitated easier absorption and utilization. Cockburn et al. [55] showed that the intestinal microbiota can degrade macromolecular polysaccharides to facilitate human absorption. However, the mechanism and the impact of fermentation on the structure of polysaccharides remain to be studied.

Excessive oxygen free radicals cause oxidative damage to the body, which promotes aging [1]. The in vitro antioxidant activity experiments showed that both FCSPs and WCSPs can scavenge DPPH·, ABTS^+^·, OH·, and O_2_^−^· radicals, and the antioxidant effect of the FCSPs was more potent than that of the WCSPs.

Both the FCSPs and WCSPs could prolong the lifespan of *C. elegans*, which was observed under standard conditions as well as under thermal or oxidative stress conditions. Studies have found that quercetin can not only delay the aging of *C. elegans*, but also increase the lifespan of *C. elegans* under stress conditions [56]. The CSPs showed a similar result, indicating that FCSPs and WCSPs can not only resist aging damage caused by nonenvironmental stress, but also improve *C. elegans* lifespan under environmental pressure. In studies of *C. elegans*, some scientists believe that both extended lifespan and reproduction require resources, which are limited in the body. Therefore, there is a balance between longevity and reproductive capacity [43,56]. The oviposition experiment showed that the FCSPs could extend the lifespan of *C. elegans* without sacrificing the oviposition of *C. elegans*, and that the FCSPs oviposited more than the WCSPs.

Previous studies indicated that the detrimental effects of free radicals on cellular components are essential causes of aging and related degenerative diseases [57]. The intracellular antioxidant enzyme system, which includes GSH-Px, SOD, and CAT, can scavenge free radicals in the body organism and modulate the redox balance in *C. elegans* in a cooperative manner. Many plant polysaccharides play a role in regulating the activity of antioxidant enzymes. Polysaccharides extracted from the roots of *Lilium davidii* var. unicolor cotton could improve antioxidant enzyme activities and decrease the MDA concentration in *C. elegans* [58]. *Pueraria lobata* polysaccharides could increase the activity of SOD and significantly reduce ROS and MDA levels to delay *C. elegans* aging under heat stress [59]. After the treatment of *C. elegans* with FCSPs and WCSPs, the activities of GSH-Px, SOD, and CAT were significantly improved with increases in the sample concentration, and the accumulation of MDA was decreased. These results indicate that the FCSPs had a better effect than the WCSPs on prolonging the lifespan of *C. elegans* by increasing the activities of the antioxidant enzymes in *C. elegans* and reducing the MDA level in vivo. The above results are consistent with the lifespan results of *C. elegans*. The superior efficacy of FCSPs may be due to the fact that CSPs were decomposed into smaller molecular weight polysaccharides or modified into polysaccharides with higher activity during the fermentation process [60].

The IIS pathway is vital for regulating the lifespan in *C. elegans*. Inhibiting the activity of the IIS pathway can improve the ability of *C. elegans* to resist oxidative and heat stress, protect or repair oxidative damage, and prolong lifespan [61].

Under stressful conditions, the entry of daf-16 into the cell nucleus in the IIS pathway increases its transcriptional activity; therefore, various aging-related genes downstream of the IIS pathway are regulated to delay *C. elegans* aging [62]. FCSPs delay aging in *C. elegans* via the IIS pathway by downregulating daf-2 and age-1, activating daf-16 transcriptional activity, and thus, promoting the expression of its target gene sod-3. The high expression of sod-3 plays an essential role in the defense against oxidative stress and is a crucial indicator of daf-16 transcriptional activity. Furthermore, skn-1 functions similarly to resist oxidative stress in *C. elegans*, so it is crucial in the stress and longevity phenotypes associated with a reduced IIS pathway [63]. In addition to daf-16, FCSPs also promoted the expression of skn-1 and its downstream target gene gcs-1 to extend the lifespan and increase the resistance of *C. elegans*. To further confirm the possible mechanism of action between FCSPs and the IIS pathway, it is necessary to use mutant strains mutated at key sites of the IIS (such as the daf-16 mutants and daf-2 mutants) as research vectors for follow-up research.

In this article, we used a fermentation method to extract CSPs. During the fermentation process, plant cell walls of herbal extracts are enzymatically degraded, followed by impregnation, to better extract secondary plant metabolites from the substrate without the need for extraction using high temperature, ultrasound, and other radiation sources [18]. Miao et al. revealed that fermentation can increase the antioxidant properties of *Auricularia auricula* polysaccharides [64]. The pyran type polymer was converted during fermentation into a furan type polysaccharide with increased antioxidant activity. Another study showed a decrease in the molecular weight and an increase in the antioxidant activity of rice bran polysaccharides after fermentation with *Grifola frondosa* fungi. The extracellular enzymes released by *Grifola frondose* may be the cause of the change in the molecular weight distribution [65]. Our results revealed that the molecular weight of the FCSPs is smaller than that of the WCSPs, exhibiting better antioxidant and antiaging effects. Therefore, we speculate that the extracellular enzymes secreted by *S. cerevisiae* during the fermentation process has led to the structure changes of the FCSPs, thus providing better antioxidant and antiaging properties. However, it is still not clear which enzyme plays a role, what is its mechanism in the fermentation process, and how different kinds of strains affect the fermentation products. In the future, the mechanism of the fermentation process should be explored at a deeper level to provide a more valuable reference for the extraction of active ingredients in fermentation technology.

## 5. Conclusions

In summary, the fermentation method is more suitable for extracting CSPs. Compared with the WCSPs, the FCSPs extracted with the fermentation method have higher antioxidant activity and can improve the ability of *C. elegans* to resist oxidative stress and heat stress, which prolong its lifespan. FCSPs mainly work through the two key routes of daf-16 and skn-1 in the IIS pathway. The above research provides the possibility for antiaging applications of FCSPs in the fields of food and medicine.

## Figures and Tables

**Table 1:** Primer list of the genes.

Gene	Direction	Primer (5′-3′)
β-actin	F	CTGAAGCCCCACTCAATCCA
	R	GCCAAGTCAAGACGGAGGAT
sod-3	F	GACGATCAACCCCTGTCGAA
	R	TACTGTTCT TCGGGGAACGC
daf-16	F	AAGCCAGGAAGGAATCCACG
	R	TTGAGTTCGGGGACGGAAAG
daf-2	F	GTAATTGGAGGCCGTTCGCT
	R	CGTGGG CACATCAATCCAGT
age-1	F	TGTGGGGACACTGACGCTG
	R	TTGGCAGTCGGTTCAGGAG
Skn-1	F	AGTGTCGGCGTTCCAGATTTC
	R	GTCGACGAATTGCGAATCA
Gcs-1	F	GTCGATGAAGCCAGATGGTTGT
	R	CGATCGTCGACACTTGCACTAA

**Table 2:** Composition of WCSPs and FCSPs.

Molar Percentage (%)	Xylose	Arabinose	Mannose	Galactose	Glucose
WCSPs	5.49	12.33	4.76	9.33	68.06
FCSPs	0.44	1.56	2.08	2.86	93.04

**Table 3:** Effect of WCSPs and FCSPs on the lifespan of *C. elegans* ($\bar{X}\pm S$).

Groups	Number of *C. elegans*	Average Lifespan (d)	Maximum Lifespan (d)
Control	90	18.02 ± 1.71	23.31 ± 0.92
WCSPs (0.2 g/L)	90	18.17 ± 0.85	24.02 ± 1.19
WCSPs (1 g/L)	90	20.61 ± 0.92	24.95 ± 1.24
WCSPs (5 g/L)	90	21.32 ± 1.08 *	25.87 ± 1.01 *
FCSPs (0.2 g/L)	90	19.76 ± 1.15	25.22 ± 1.19
FCSPs (1 g/L)	90	21.53 ± 0.98 **	26.47. ± 1.58 *
FCSPs (5 g/L)	90	22.58 ± 1.21 **	27.09 ± 1.03 **

Note: Values are mean ± SD, *n* = 3. Compared with control, * *p* < 0.05; ** *p* < 0.01.

**Table 4:** Effect of WCSPs and FCSPs on the heat resistance time of *C. elegans* ($\bar{X}\pm S$).

Groups	Average Heat Resistance Time (h)	Average Lifespan Increase (%)	The Longest Heat Resistance Time (h)
Control	8.10 ± 0.37	—	12.00 ± 0.81
WCSPs (0.2 g/L)	8.60 ± 0.29	6.17	12.67 ± 0.94
WCSPs (1 g/L)	9.19 ± 0.27 **	13.45	13.67 ± 0.47 *
WCSPs (5 g/L)	10.93 ± 0.69 **	34.93	14.33 ± 0.47 **
FCSPs (0.2 g/L)	9.35 ± 0.36 **	15.43	14.67 ± 0.94 **
FCSPs (1 g/L)	10.41 ± 0.58 **	28.51	18.33 ± 0.47 **
FCSPs (5 g/L)	11.21 ± 0.71 **	38.39	18.67 ± 0.94 **

Note: Values are mean ± SD, *n* = 3. Compared with control, * *p* < 0.05; ** *p* < 0.01.

**Table 5:** Effect of WCSPs and FCSPs on the oxidative resistance time of *C. elegans* ($\bar{X}\pm S$).

Groups	Average Oxidative Resistance Time (h)	Average Lifespan Increase (%)	The Longest Oxidative Resistance Time (h)
Control	7.04 ± 0.17	—	11.67 ± 0.47
WCSPs (0.2 g/L)	7.42 ± 0.41	5.39	12.33 ± 0.47
WCSPs (1 g/L)	8.59 ± 0.22 **	22.02	13.67 ± 0.94 *
WCSPs (5 g/L)	9.38 ± 0.59 **	33.23	14 ± 0.81 **
FCSPs (0.2 g/L)	7.67 ± 0.28 *	8.94	12.67 ± 0.47
FCSPs (1 g/L)	9.51 ± 0.45 **	35.08	14.33 ± 0.47 **
FCSPs (5 g/L)	9.84 ± 0.24 **	39.77	15.67 ± 0.94 **

Note: Values are mean ± SD, *n* = 3. Compared with control, * *p* < 0.05; ** *p* < 0.01.

## Data Availability

The data are available from the corresponding authors upon reasonable request.
